# Affinity and Pseudo-Affinity Membrane Chromatography for Viral Vector and Vaccine Purifications: A Review

**DOI:** 10.3390/membranes13090770

**Published:** 2023-08-30

**Authors:** Keven Lothert, Michael W. Wolff

**Affiliations:** Institute of Bioprocess Engineering and Pharmaceutical Technology, Department Life Science Engineering, University of Applied Sciences Mittelhessen (THM), 35390 Giessen, Germany

**Keywords:** virus purification, virus-like particles, sulfated cellulose, affinity ligands, glycosaminoglycans, convective-flow media, downstream processing

## Abstract

Several chromatographic approaches have been established over the last decades for the production of pharmaceutically relevant viruses. Due to the large size of these products compared to other biopharmaceuticals, e.g., proteins, convective flow media have proven to be superior to bead-based resins in terms of process productivity and column capacity. One representative of such convective flow materials is membranes, which can be modified to suit the particular operating principle and are also suitable for economical single-use applications. Among the different membrane variants, affinity surfaces allow for the most selective separation of the target molecule from other components in the feed solution, especially from host cell-derived DNA and proteins. A successful membrane affinity chromatography, however, requires the identification and implementation of ligands, which can be applied economically while at the same time being stable during the process and non-toxic in the case of any leaching. This review summarizes the current evaluation of membrane-based affinity purifications for viruses and virus-like particles, including traditional resin and monolith approaches and the advantages of membrane applications. An overview of potential affinity ligands is given, as well as considerations of suitable affinity platform technologies, e.g., for different virus serotypes, including a description of processes using pseudo-affinity matrices, such as sulfated cellulose membrane adsorbers.

## 1. Introduction

**Figure 1:**
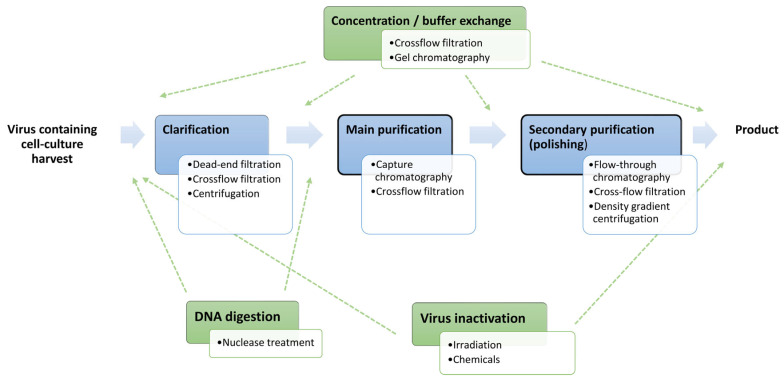
Typical process for the purification of cell culture-derived biological nanoplexes, such as viruses. Essential steps are initial clarification followed by purification using at least two orthogonal techniques, which form the basic backbone of the process (blue). Additional unit operations are optional (green) and depend on the actual product, e.g., whether the viral product should be active or inactivated, whether a buffer exchange or concentration of the product is desired at a certain point in the process, or whether an additional degradation of the contained DNA is required (usually the case). These optional procedures can be integrated flexibly, and typical positions are indicated by the dashed arrows. Affinity chromatography is often an integral part of purification and is performed as a primary or secondary purification (bold framed).

The production of viruses and virus-like particles (VLPs) for biopharmaceutical uses, such as vaccines or viral vector applications, requires an extensive product purification to reduce safety risks for patients. Depending on the production system of the particular product, the composition of contaminants in the starting material can vary [1,2]. In general, these contaminants include cell debris from the production host cells, including host cell-derived DNA and proteins, as well as additives supplemented during the actual production process, e.g., surfactants, antibiotics, or serum proteins. In addition, defective product particles and other non-product particles, such as exosomes or extracellular vesicles, may occur. Due to this complexity, the downstream purification process (DSP) that is employed must be highly versatile. While many different purification techniques can be used, chromatography is an important unit operation for virus processing, along with filtration-based applications (Figure 1) [3].

When focusing on chromatographic approaches, a variety of techniques are available that allow for a tailored method development depending on the characteristics of the target product. Possible approaches include charge- and pH-driven principles using ion exchange chromatography [4,5,6,7,8,9,10,11], hydrophobic interaction chromatography [12,13,14,15], size-dependent separation principles such as size exclusion [16,17,18,19] or steric exclusion chromatography [20,21,22,23], and affinity applications [24,25]. In addition, combined approaches using mixed-mode resins have recently been described, with restricted access media (mixed-mode size exclusion chromatography) resins as the most promising option for separating large virus particles from smaller DNA and protein contaminants [6,26,27,28]. These methods differ in their specificity and selectivity for separating product viruses or VLPs from process-related contaminants, namely host cell-derived proteins and DNA or from other particles produced alongside with the products, e.g., extracellular vesicles [29,30]. Particularly the latter requires a sufficient selectivity of the method, as these particulate contaminants share similar characteristics with the product [31,32,33]. Hence, size- or charge-dependent approaches are usually not sufficient and sometimes only the highly specific affinity interaction is able to achieve a chromatographic separation [34]. Additionally, the choice of one or the other method depends not only on the physicochemical properties of the product but also on its position in the downstream purification scheme. For example, size-exclusion chromatography typically requires a small sample-to-column volume ratio (about 5%) and is, therefore, ideally performed at a later stage in the process with concentrated feed material [35]. On the other hand, well-adapted ion exchange applications, and even more so affinity matrices, are capable of isolating a specific target from a complex feed suspension and can, therefore, be used earlier in the process as a capture step (Figure 1). There are several publications that focus on general purification schemes for viruses and VLPs [3,36,37,38,39,40,41,42,43], on chromatographic approaches in general [44], or more specifically on selected chromatographic separation principles such a as steric exclusion chromatography [23], ion exchange chromatography, or affinity techniques [24,25,45]. However, the latter is often performed using resin-based columns with porous particle beads. The superiority of convective flow materials, such as monoliths [46,47,48] and membranes [49,50], over traditional particle resins has been widely discussed and will not be considered in detail here. In short, the advantages of convective flow columns are particularly evident in the purification of larger products such as viruses and VLPs and include higher binding capacities, higher possible flow rates, and thus an increased productivity [51]. With regard to convective flow materials, the use of monoliths is often associated with higher costs for the chromatographic units and more cumbersome handling, due to their size and their cleaning and regeneration limitations [52,53]. A major drawback often described for membrane chromatography applications is a lower resolution and the reduced dynamic and equilibrium binding capacity, which might limit their application for capture purifications. However, these limitations are most distinct for small target molecules, e.g., proteins, and become negligible for larger products, such as viruses and VLPs [49,51,54,55,56]. Additionally, membranes are particularly suitable for single-use applications that completely eliminate the need for rigorous regeneration procedures. For affinity chromatography, however, the cost-driving factor is usually the immobilized affinity ligand, depending on its source or structure, making single-use approaches uneconomical for most chromatographic methods where proteins or nucleic acids are applied as ligands.

Here, we summarize the modern membrane-based affinity and pseudo-affinity purifications of viruses and VLPs while also briefly covering the history of bead-based resin affinity purifications for these products in general. We also provide a brief overview of the methods, which are used to identify affinity ligands as well as the relevant decision criteria for their selection. In addition, we indicate which types of ligands are suitable for use with membranes and give a brief outlook on the future prospects of affinity chromatography using membranes.

## 2. Development of Affinity Purifications for Viruses and VLPs and Currently Established Applications

**Figure 2:**
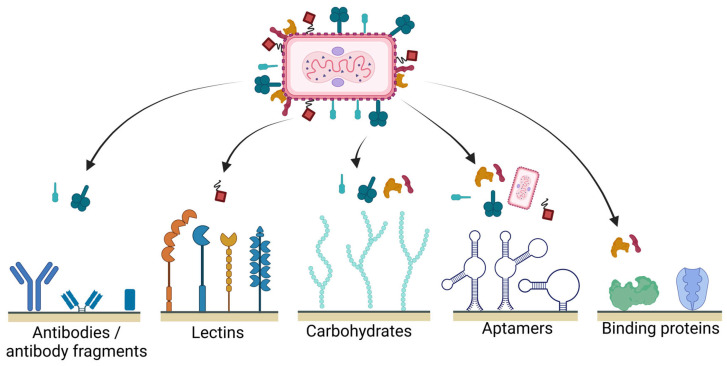
Types of affinity ligands suitable for virus purification. The virus is shown schematically, and is not based on an actual native virus, to illustrate possible surface structures. (Created in Biorender.com, agreement number: KB25MV2G5L).

Originally, affinity purifications were established for biopharmaceutical proteins, especially for antibodies, and were based on a classical antibody–antigen interaction, where the antigen is immobilized on a stationary phase. A technology that has been established as a platform purification approach for various antibody purifications are the Protein A, G, or L affinity resins, which represent the most widely used affinity matrix materials on the market, taking into account several modifications and optimizations of the ligand composition over the years [45]. For viral products, the identification of a comparable ligand that is able to bind to a broad portfolio of different viruses or VLPs is not trivial. These biological nanoplexes are composed of a complex surface composition, including different glycoproteins and attached lipids. Due to this heterogeneity, not only antibodies and antibody fragments but also other binding proteins, carbohydrates, lectins, or aptamers can be considered as ligands (Figure 2). Unfortunately, the identification of suitable binding interaction targets is not trivial. Applicable ligands can be revealed either by experimental screening, by an evaluation of ligand libraries with surface plasmon resonance or biolayer interferometry applications [57,58], or theoretically by molecular modeling [59] and also by considering the available literature. For the selection of optimal ligands, a number of different aspects need to be considered. One of these aspects is related to differences in the surface structure of the viruses in changing subtypes, such as in influenza [60,61,62]. In this case, the composition of the vaccine is subject to recurring annual adjustments. Another challenge is the variation in the composition of different subtypes of adeno-associated viruses (AAV) [63,64]. Hence, ligands must be specific for the virus to be produced, but in order to ensure a robust process control for the different subtypes, a conserved region in a common protein over a wide range of subtypes needs to be targeted. Another requirement for potential affinity ligands is the avoidance of ligand-related toxic effects. One example is the use of lectins as ligands (Figure 2) [65], which are highly dependent on the selected production host cell, and thus allow a specific affinity interaction [66,67,68]. However, several lectins are described to cause severe toxic effects in humans [69], rendering their application in biopharmaceutical production processes inconvenient. The use of other carbohydrates for affinity interactions, such as glycans, can avoid these issues [70], hence a careful ligand selection preceding the process set-up is essential. An overview of suitable potential affinity ligands for virus purifications is depicted in Figure 2.

For various viruses, individual ligands have been identified and immobilized onto a stationary phase backbone, often consisting of resin particle beads of silica, cellulose, or dextran. Reports of various applications have been reviewed in previous publications [24,25]. A recent example is the generation of an agarose-based affinity column for the selective retention of lentiviral vectors, which were pseudotyped with a vesicular stomatitis virus glycoprotein (VSV-G) envelope [71]. In this study, the group performed a ligand screening, using surface plasmon resonance sensors to generate a scalable affinity column afterwards.

As variations in the composition of the product (virus, VLP) can alter its binding interaction with the column, it is desirable to use ligands that are suitable for a wider range of targets. With this in mind, it is possible to select viral surface structures that are present on all viruses of the same class regardless of the viral genotype, such as the gp64 protein found on many different baculoviruses [72], or the use of antibody fragments suitable for various AAV subtypes, as applied in the AVB Sepharose, or POROS CaptureSelect columns [64,73,74]. However, if a different virus were to be used, method development and ligand screening would have to start anew. The basis for an evaluation of suitable ligands can be an assessment of virus–cell interactions occurring during a virus infection [75,76,77,78,79,80]. These are often similar between various viral subtypes and also for different viruses. An attachment of viruses to their respective host cells precedes the actual infection and is usually mediated by virus–receptor interactions, cellular glycosaminoglycans (GAGs), or sialic acid containing carbohydrates, e.g., glycans [78,81,82]. A current platform technology that has been found to be suitable for various viruses and some VLPs is based on glycosaminoglycans (GAGs), such as heparin and heparan sulfate, respectively [83,84,85]. As many viruses have a natural affinity for the highly sulfated linear polysaccharide chain of these GAGs [86,87], a selective product retention is possible while avoiding a co-purification of the major contaminant DNA and protein. Several reports have described the successful purification performance of heparin columns, e.g., for baculoviruses [88], retroviral vectors [89], foot-and-mouth disease virus [90], modified vaccinia ankara MVA virus [91], as well as for the separation of VLPs and extracellular vesicles [92,93], to name only a few. However, heparin as a ligand is of animal origin, making it expensive to implement, limited in availability, and questionable for a use in purification processes under good manufacturing practice (GMP) conditions for the production of human pharmaceuticals. Recently, the recombinant generation of bioengineered heparin has been described using *Pichia pastoris* as a production host. [94]. While this avoids the problems associated with animal-derived heparin, such as contamination, impurities, and regulatory issues, the application of heparin remains cost-intensive. Another issue is the high diversity of the individual heparin ligands. These may vary in the degree of sulfation and in the molecular weight of the individual heparin chains. This particularly accounts for resins obtained from different vendors, but batch-to-batch variations are also observable for different lots of the same manufacturer. For these reasons, affinity columns are often replaced by orthogonal purification techniques such as ion exchange chromatography in large-scale production. Therefore, although heparin affinity purification is widely used, it is highly unlikely to be adapted for membrane processes, as disposable membrane columns would make DSP uneconomical.

Another approach to avoid heparin and to, nevertheless, use an affinity platform purification approach is to use ligands that mimic the properties of heparin, namely the highly sulfated polysaccharide chain [95]. For resin-based chromatography, dextran sulfate and sulfated cellulose matrices have become an established alternative, with several commercial products available on the market [45]. As these matrices are chemically sulfated, making the backbone of the stationary chromatographic phase to the actual ligand, the ligand distribution on the surface of the stationary phase can be adjusted homogenously, and a modification of the actual ligand density is relatively straightforward. The purification performance of these heparin analogues, i.e., dextran sulfate or sulfated cellulose, has been shown to be effective for viruses with heparin affinity and has been described for the purification of the MVA virus [91,96], the influenza virus [97], and the AAV [98,99]. For the influenza virus, the use of sulfated cellulose beads has been estimated to significantly reduce the overall matrix cost compared to specific ligands purified from natural sources such as heparin or lectins, although the authors did not provide detailed cost calculations [65,100].

In addition to bead-based particle resins, monolithic backbones are often used for an affinity and pseudo-affinity purification of viruses. Examples from recent years include the preparation of monolithic hydrogel columns with heparin ligands for the DSP of the enterovirus 71 [101], the purification of the cowpea chlorotic mottle virus with custom-made monoliths using peptide aptamers as ligands [102], or the separation of empty capsids from AAV preparations employing metal affinity [103]. Earlier examples of these applications have been summarized in previous reviews [24,25]. Although the use of monolithic columns avoids some of the disadvantages of resin particles, by providing convective flow properties and improved utilization of the available binding capacity, the fabrication of a monolithic column is costly, making it unattractive for single-use applications. In addition, the cleaning and regeneration of these monoliths can be a major challenge, as described previously [15,52]. Finally, the handling of monolithic columns is cumbersome, especially for large-scale applications and when aseptic handling and additional sterilization procedures are required, considering the size and weight of commercially available process-scale (4–8 L) monoliths. Recent improvements in this area include the development of monolith-like particle stationary phases. These combine the handling properties of bead-based resins with the convective flow of monoliths. An example of such a stationary phase is the preparation of cellulose monolith-like particles, modified with dextran sulfate, for a pseudo-affinity purification of influenza viruses [104]. In this study, the dynamic binding capacity was increased 5–11 times compared to commercial bead-based resins, and the column could be reused, if desired, for at least 10 cycles, including an appropriate cleaning procedure. The use of monolith-like particles indicates an improvement of some of the abovementioned drawbacks affecting the use of monoliths, but their production remains cumbersome, particularly for the larger scales, compared to the manufacturing of membranes.

## 3. Affinity and Pseudo-Affinity Membranes

The advantages of membrane processes over classical bead-based resins and also as an alternative to monolithic columns for the chromatographic purification of biological nanoplexes have been widely discussed in previous reviews [3,44]. In summary, the use of membrane-based stationary phases is able to avoid most of the disadvantages of particle resins and monoliths described in the previous section. In return, suitable membrane materials have been developed for many different chromatographic separation principles and are commercially available and widely used for virus and VLP purification, e.g., anion exchange [6,28,93,105,106,107], cation exchange [108,109], or hydrophobic interaction [14,108]. Affinity purifications, on the other hand, are typically highly customized applications that require tailor-made stationary phases. As a result, these materials have a limited range of applications, and their production for large-scale applications is currently only economically viable for seasonal viral vaccines, common viral vectors, and established oncolytic viruses. Consequently, affinity chromatography for other viral targets that require GMP process-scale purifications are only considered to a limited extent. Thus, it is questionable whether it makes sense to push the development of such matrices on a small scale for other applications. Nevertheless, many membrane-based affinity purifications of viruses are performed with custom-made stationary phases, resulting in promising product recoveries and impurity depletions by immobilizing metal ions [110,111], lectins [112], heparin [91,96], or by sulfating the cellulose membrane backbone for pseudo-affinity purifications [96,113] (Table 1). While at least four GMP-compliant products are available for bead-based resins that are suitable for a pseudo-affinity platform purification [45] (see Section 2), only the sulfated cellulose membrane adsorber is commercially available, but it is not GMP-suitable for this kind of application. Sulfated cellulose membranes mimic heparin affinity, as described above, and bind all types of viruses with a heparin affinity [114]. Although this membrane adsorber is theoretically capable of purifying a wide variety of viruses, the membrane properties, such as the ligand density and the pore size distribution, were initially optimized for the purification of influenza viruses [42,115]. In return, the product yields highly varied for the purification of different viruses and VLPs using this method (Table 1).

An overview of previous studies, focusing on affinity purifications using membranes as stationary phases, is shown in Table 1. The available data suggest that research efforts are currently limited to comparatively few different ligands, mainly focusing on laboratory-scale (<200 mL) purifications. Further research and product development would be desirable, especially considering the continuously increasing pharmaceutical importance of virus-based treatments [116]. Within the scope of the purification methods here, and with reference to membrane-based chromatography affinity with its high specificity and high binding capacities by membrane adsorbers, it generally allows relatively high virus recoveries of up to 100%, while at the same time providing an efficient impurity removal. In terms of this impurity removal, the separation of the product from contaminating DNA is particularly important. In several reports, more than 90%, and occasionally up to 99.9%, of the DNA was removed. For non-affinity chromatographic applications, a similar purification performance cannot be achieved due to co-elution of contaminating DNA, especially in ion exchange processes [4,108], but to a limited extent also for other applications, such as steric exclusion chromatography [117]. This makes affinity chromatography, and especially the use of scalable disposable materials with convective flow, a valuable tool for the DSP of larger biological nanoplexes.

While sulfated cellulose membrane adsorbers have been rigorously evaluated, a further expansion of the membranes’ product portfolio, in terms of pore size distribution and ligand densities, would increase the attractiveness of this pseudo-affinity method for the purification of a diversity of viruses on varying scales. A different approach to broad platform applications could be a focus on individual viruses that are currently of high pharmaceutical relevance, such as the adeno viruses and AAV, retroviruses, herpes simplex viruses, or hepatitis viruses, to name a few. It would be highly desirable to promote the development of scalable affinity membranes for these viruses as has been done recently for lentiviral vectors using a bead-based resin [71]. A comparable approach was made for resin column systems for the purification of AAV [45,98] and could be feasible for membranes as well. Further developments could overcome the limitations of currently used affinity membranes, which are usually custom-made and not suitable for commercial or GMP processes.

**Table 1:** Overview of affinity membrane applications for virus and VLP purification processes.

Target	Ligand/Membrane Material	Commercially Available	Processed Feed Volume (Scale)	Yield/Recovery	Impurity Depletion		Reference
		[Yes or No]	[mL]	[%]	Protein [%] ^1^	DNA	
Adenovirus	Zn^2+^/cellulose	Yes (Sartorius)	200	87	<25 pg mL^−1^	<0.3 ng mL^−1^	[110]
Hepatitis C virus	Sulfated cellulose/cellulose	Yes (Sartorius)	10	50	Not determined	78%	[21] ^2^
Influenza A virus	Euonymus europaeusLectin/cellulose	No	20	108	69%	99%	[112]
	Zn^2+^/cellulose	Yes (Sartorius)	50	64	93%	74%	[111] ^3^
	Sulfated cellulose/cellulose	Yes (Sartorius)	10	73–94	57–84%	68–99%	[113] ^4^
	Sulfated cellulose/cellulose	Yes (Sartorius)	70	80	71%	97.5%	[107]
	Sulfated cellulose/cellulose	Yes (Sartorius)	<10	57	1.2 ± 6 0.02 ng_prot_ HAU^−1^	5.1 ± 0.2 pg_DNA_ HAU^−1^	[115] ^5^
	Sulfated cellulose/cellulose	Yes (Sartorius)	<10	64	0.013 mg_prot_ µg_HA-1_	0.0038 µg_DNA_ µg_HA_^−1^	[42]
	Sulfated cellulose/cellulose	Yes (Sartorius)	10 per cycle ^6^	67.4	67.4	99.8	[118]
Influenza VLPs	Sulfated cellulose/cellulose	Yes (Sartorius)	<10	80	89%	80%	[42]
Orf virus	Sulfated cellulose/cellulose	Yes (Sartorius)	10	34–54	>99%	20–95%	[108] ^7^
Vaccinia virus/Modified Vaccinia Ankara virus	Heparin/cellulose	No	<10	56	99%	76%	[91]
	Sulfated cellulose/cellulose	Yes (Sartorius)	20	65	99%	90%	[91]
	Heparin/cellulose	No	<10	68	99.9%	80%	[96]
	Sulfated cellulose/cellulose	Yes (Sartorius)	<10	75	99.9%	95%	[96]

^1^ If no relative values for depletion were stated, the final impurity concentration after the purification is given. ^2^ The pseudo-affinity purification was performed as a secondary/final purification step. ^3^ Prior to optimizing the method for Zn^2+^, six other metal affinity ligands were screened (Ni^2+^, Cu^2+^, Al^3+^, Mn^2+^, Ca^2+^, and Fe^3+)^. ^4^ Three different Influenza A strains were evaluated; thus, recoveries and impurity depletions vary. ^5^ HA and HAU refer to hemagglutination units, a measure for the quantification of influenza viruses. ^6^ The process was performed in a continuous periodic counter current operation (3 sections/cycles over 10 loops). ^7^ Pseudo-affinity was evaluated as a capture step and for secondary purification. The product recoveries and impurity depletions varied accordingly.

In principle, the use of membrane materials supports the implementation of single-use applications, which reduce cleaning and validation efforts as well as potential cross-contamination [51,54,119]. However, for many affinity ligands, single use is not a viable option, due to the cost of the ligands themselves. Furthermore, affinity ligands are generally highly target-specific, limiting a broader commercialization and restricting such membrane applications to the laboratory scale. To overcome these limitations, platform technologies for different virus classes are highly preferred. For particle resins, different platform technologies have been described, including highly pharmaceutically relevant products, such as AAV and different AAV subtypes (see Section 2). While AAV is a rather small virus particle and thus benefits less from the convective flow approach [120,121], especially for larger viruses, the adaption to membrane processes seems promising. A transfer of the ligand composition from bead particles to membranes stands to reason but has not been implemented into the product portfolio of many manufacturers yet.

The only commercially available membrane material suitable for a purification platform is the heparin-mimicking sulfated cellulose membrane adsorber, which allows for a pseudo-affinity purification of various viruses and VLPs. However, the possible implementation in a DSP process is limited not only by the requirement for the product affinity but also by the size of the product due to the rather narrow pore size (about 0.8 µm) of these membranes.

## 4. Conclusions

Currently, many chromatographic applications for an affinity purification of viruses and VLPs using affinity ligands are based on resin or gel column backbones, and several different column types are commercially available. However, due to the limited process productivity of these columns as a result of diffusion-limited mass transport, convective flow materials such as monoliths and membranes are a promising choice for a wide range of these applications.

To enable the robust single-use affinity membrane purification of large biopharmaceuticals, such as viruses, further research is essential and will become increasingly important in the future due to the rising number of medical applications of virus-based pharmaceuticals and the need for flexible and, at the same time, specific purification approaches. Ideal ligands for such pseudo-affinity purifications must (i) be specific enough to separate the target from contaminating DNA and proteins, as well as from other particles; (ii) be unaffected by variations in the composition of the targets and ideally suitable for a range of different virus types or at least for genotypic variations of individual (pharmaceutically important) viruses (platform application); (iii) allow for mild elution conditions to not affect the viral activity or the stability of the virus-like particles; and (iv) allow for an economic production and immobilization on the membrane surface. The last point, in particular, is crucial, as otherwise single-use applications are not financially feasible, thus severely compromising one of the major advantages of membrane chromatography applications.

## Data Availability

No new data were created or analyzed in this study. Data sharing is not applicable to this article.
